# Socioeconomic Assessment and Impact of Social Security on Outcome in Patients Admitted with Suspected Coronary Chest Pain in the City of Salta, Argentina

**DOI:** 10.1155/2013/807249

**Published:** 2013-05-29

**Authors:** Ricardo A. León de la Fuente, Patrycja A. Naesgaard, Stein Tore Nilsen, Leik Woie, Torbjoern Aarsland, Harry Staines, Dennis W. T. Nilsen

**Affiliations:** ^1^Department of Cardiology, Stavanger University Hospital, Postboks 8100, 4068 Stavanger, Norway; ^2^Institute of Medicine, University of Bergen, Postboks 7804, 5020 Bergen, Norway; ^3^Cardiology Research Institute, Catholic University of Salta, España 311, A4400ANG Salta, Argentina; ^4^Department of Research, Stavanger University Hospital, Postboks 8100, 4068 Stavanger, Norway; ^5^Sigma Statistical Services, School Road, Balmullo KY16 0BJ, UK

## Abstract

Low socioeconomic status is associated with increased mortality from coronary heart disease. We assessed total mortality, cardiac death, and sudden cardiac death (SCD) in relation to socioeconomic class and social security in 982 patients consecutively admitted with suspected coronary chest pain, living in the city of Salta, northern Argentina. Patients were divided into three socioeconomic classes based on monthly income, residential area, and insurance coverage. Five-year follow-up data were analyzed accordingly, applying univariate and multivariate analyses. At follow-up, 173 patients (17.6%) had died. In 92 patients (9.4%) death was defined as cardiac, of whom 59 patients (6.0%) were characterized as SCD. In the multivariate analysis, the hazard ratios (HRs) for all-cause and cardiac mortality in the highest as compared to the lowest socioeconomic class were 0.42 (95% confidence interval (CI), 0.22–0.80), *P* = 0.008, and 0.39 (95% CI, 0.15–0.99), *P* = 0.047, respectively. Comparing patients in the upper socioeconomic class to patients without healthcare coverage, HRs were 0.46 (95% CI, 0.23–0.94), *P* = 0.032, and 0.37 (95% CI, 0.14–1.01), *P* = 0.054, respectively. In conclusion, survival was mainly tied to socioeconomic inequalities in this population, and the impact of a social security program needs further attention.

## 1. Introduction

In addition to the well-known risk factors for cardiovascular disease, such as hypertension, diabetes, cigarette smoking, hypercholesterolemia, obesity, and a sedentary lifestyle [1], socioeconomic status may also have an impact on prognosis [2].

As socioeconomic inequalities vary between countries and regions, they need to be accounted for in risk assessment within communities with marked social gradients.

To our knowledge, socioeconomic inequalities and their associations with cardiovascular mortality have not been studied in Salta, northern Argentina.

According to official data [3] 7.9% of the population in the Salta region is unemployed, but a wider definition than the one used would indicate an incidence of 29.3% (local data attached to the official data), and 60% are without a health security program (Table 1) [3].

The city of Salta has two large public hospitals, one for adults (405 beds) and one for obstetrics and pediatrics. They provide comprehensive care for patients in all medical and surgical specialties, but high-cost medical treatment is not widely available in the province of Salta.

In the city of Salta there are also eleven private clinics with a total number of beds similar to that of the public hospital. Catheterization laboratory facilities are available at the public hospital and in five of the private hospitals. ST-elevation myocardial infarction (STEMI) patients are offered primary percutaneous angioplasty, whereas thrombolytic treatment is rarely used. Although, technologically advanced treatment for coronary heart disease (CHD) may be limited, in-hospital medical treatment is offered according to international guidelines.

The majority of patients in the public hospitals are not covered by a social security program. Patients without healthcare coverage have limited access to out-of-hospital attention, including post-discharge follow-up.

In this study our main objective was to examine 5-year survival according to socioeconomic status of patients admitted with suspected coronary chest pain in the city of Salta, in northern Argentina. Endpoints consisted of all-cause mortality, cardiac death, and sudden cardiac death (SCD). In addition, we evaluated the impact of a social security program on survival. 

## 2. Methodology

### 2.1. Study Design and Patient Population

The details of the ARgentinean Risk Assessment Registry in the Acute Coronary Syndrome; the “ARRA-RACS Study” (ClinicalTrial.gov NCT01377402), have been published previously [4, 5]. Briefly, the study was performed at nine hospitals in the city of Salta, northern Argentina, including the public hospital and eight of the eleven private clinics. We included 982 patients hospitalized consecutively with chest pain and suspected acute coronary syndrome (ACS), from December 2005 to January 2009.

The primary outcome was 5-year all-cause mortality from the time of inclusion. Secondary outcomes included cardiac death and SCD, as previously described [4]. Main exclusion criteria were age <18 years, unwillingness or incapacity to provide informed consent, and prior inclusion in the present study. Baseline characteristics and clinical parameters of the total population have previously been described [4].

In order to assess the socioeconomic status, participants completed a case report form that included their monthly income, residential area, and type of insurance coverage. Socioeconomic information was captured for all patients.

A socioeconomic model was used to divide these patients into three groups: low, middle, and high socioeconomic level.

Participants were invited to an office visit at 12 months and interviewed over telephone at 30 days, 6 months, and 24 months and every year up to 5 years. Family members, neighbors, and the national registry department were contacted to obtain relevant information regarding relocations and cause of death.

The study was approved by the Ethics Committee at the Board of Medical School of Salta and at the local institutions [4] and was conducted in accordance with the Helsinki Declaration of 1971, as revised in 1983. The study was also approved by the Norwegian Authorities and was monitored by Stavanger Health Research, Stavanger, Norway.

Written informed consent was obtained from all patients.

### 2.2. Socioeconomic Model

We divided the total population into three groups, based on the following variables. The first variable was the monthly income of the patient or patient's provider, scored from 1 to 4; patients earning less than 2000 Argentinean pesos a month were assigned a score of 1, those with a monthly income between 2000 and 4000 were given a score of 2, those between 4000 and 10000 were given a score of 3, and finally those earning more than 10000 pesos a month were assigned a score of 4. The monthly salary was affirmed by the Institute of Business Information of Salta (http://www.escudosalta.com.ar/), a non-governmental and non-profit organization. Argentina has experienced an increasing rate of inflation. However, salaries were adjusted according to an annual estimate of inflation and were reasonably balanced during the years of inclusion. The second variable described the health insurance, also graded from 1 to 4; subjects without insurance coverage were assigned a score of 1, subjects on a retirement social security program were scored as 2, state employees and union associated employees as 3, and finally patients with private health insurance as 4. The third variable was the residential area, graded from 1 to 4 according to location and facilities, ranging from poor to wealthy areas. Slums with housing conditions lacking basic elements were assigned a score of 1, suburbs closest to the city center were scored 2, households located downtown were given score 3, and finally wealthy areas were assigned a score of 4.

We added the grading of each variable, obtaining a socioeconomic level for each subject, and according to this level they were divided into three categories: low social class (3–5 points), middle class (6–8 points), and upper class (9–12 points). Accordingly, 147 out of 155 individuals without social security were found in category one, whereas all subjects in category three had a social health care program. Occupation was not included as an individual variable in this socioeconomic model, as it is closely related to income in this community.

### 2.3. Definition of Employment

All persons with at least one occupation and one hour of paid work during the last week prior to the census were defined by the central government as employed, rendering an unemployment rate of 7.9% [6]. However, local data in the same document indicate an unemployment rate of 29.3% (not defined) [3]. Within the working period of life (18 to 65 years), the ratio between the employment of males and that of females in the northern region of Argentina is 0.54 according to the Argentinean census of 2010 [6].

### 2.4. Blood Samples and Laboratory Measurements

Blood samples were drawn immediately following admission. A second blood sample for repeated troponin T (TnT) was drawn 6 hours following the first sample. Baseline laboratory data included measurements of 25-hydroxyvitamin D [25(OH)D (D represents D_2_ and D_3_)], TnT, high sensitivity C-reactive protein (hsCRP), glucose and lipids in serum, B-type natriuretic peptide (BNP) measured in ethylene diamine tetraacetic acid plasma, and estimated glomerular filtration rate (eGFR) (calculated by Modification in Diet in Renal Disease formula).

### 2.5. Patient Medical Care

In-hospital medical treatment was offered according to international guidelines, although coronary revascularization was not as available as internationally recommended, due to the uneven city distribution of interventional resources. Thirty-one percent of the TnT positive population was classified as STEMIs, and within this group only 42% were treated with primary percutaneous coronary intervention (PCI). Furthermore, thrombolytic therapy in this region of Argentina is uncommon and was not applied in our patient cohort [4]. Patients without healthcare coverage are included in the public healthcare system, but due to the limited resources they do not receive optimal postdischarge care. In the City of Salta there are 75 cardiologists to cover a population of 536 113 (Table 1), but these doctors are mainly private specialists and do not cover public health care.

### 2.6. Statistical Analysis

Patients were divided into three socioeconomic classes according to the socioeconomic model. Approximately normally distributed variables were given as mean and standard deviation. The Chi-square test for association was applied between the categories and categorical variables at baseline. The one-way ANOVA was used to test for equality of means of scale variables (e.g., age) amongst the categories. The hazard ratios (HRs) are presented with 95% confidence interval (CI). Stepwise Cox multivariable proportional hazards regression models with total death, cardiac death and SCD as the dependent variable, and other variables as potential independent predictors (listed below) were fitted. To examine the differences in prognosis between subjects in the upper socioeconomic class and those in the lowest, we adjusted for gender and age only, as well as for several baseline variables including gender, age, smoking, hypertension, index diagnosis, diabetes mellitus (DM), congestive heart failure (CHF) (defined as Killip-Kimball class [7] at admission; patients in class 2 to 4 were classified as CHF patients and patients in class 1 were classified as non-CHF), history of previous CHD (i.e., history of either angina pectoris, myocardial infarction, coronary artery bypass graft, or PCI), hypercholesterolemia/use of statins, TnT > 0.01 ng/mL, eGFR, hsCRP, BNP, 25(OH)D, body mass index (kg/m^2^), months of sampling, and beta blockers prior to enrolment. The Kaplan-Meier product limits were used for plotting the times to event and the log-rank test used to compare survival curves across categories. The statistical analyses were performed using the statistical package SPSS version 19.0. All tests were two sided with a significance level of 5%.

## 3. Results

A total of 982 patients (588 men and 394 women) were enrolled in the ARRA-RACS study. No patients were lost to follow-up. Mean age in the total population was 62.2 ± 13.4 years. Baseline characteristics according to the three socioeconomic classes and patients with and without social security coverage are presented in Tables 2 and 3, respectively.

After a follow-up period of 5 years, 173 patients (17.6%) had died. In 92 patients (9.4%) death was defined as cardiac, of whom 59 patients (6.0%) were characterized as SCD.

In our multivariate analyses, the same trends were observed by adjusting for gender and age only (Table 4(a)), as for applying the entire set of variables in the model (Table 4(b)), but differences were strengthened for TnT positive patients with the latter model.

**Figure 1:**
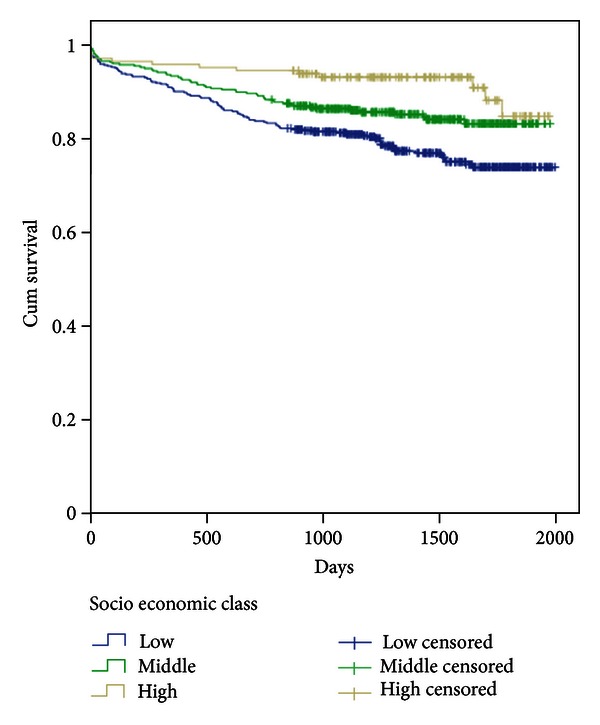
Kaplan-Meier plots depicting total mortality during 5-year follow-up in individual socioeconomic groups, based on the total patient material.

**Figure 2:**
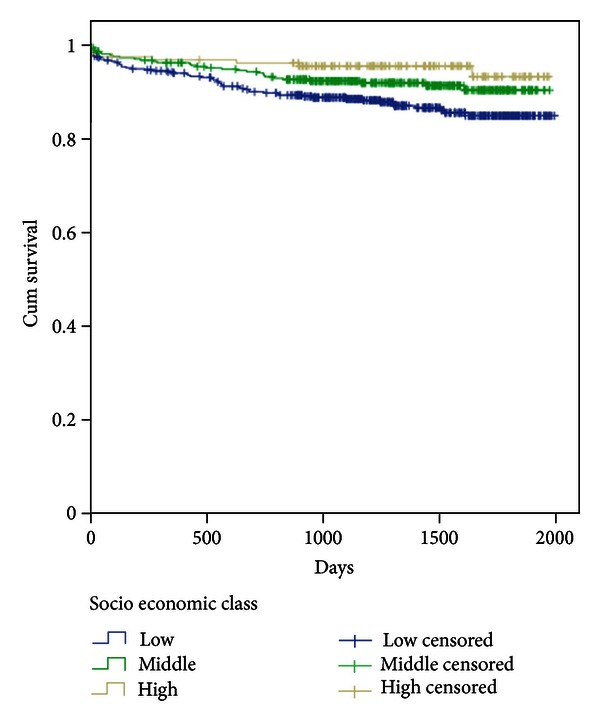
Kaplan-Meier plots depicting cardiac mortality during 5-year follow-up in individual socioeconomic groups, based on the total patient material.

In the multivariate analysis adjusting for several variables (Table 4(b)), the HRs for all-cause mortality and cardiac death in the highest socioeconomic class as compared to the lowest were 0.42 (95% CI, 0.22–0.80), *P* = 0.008, and 0.39 (95% CI, 0.15–0.99), *P* = 0.047, respectively, whereas the results were not significant for SCD. Kaplan-Meier survival curves for all-cause mortality and cardiac death comparing highest with lowest socioeconomic class are presented in Figures 1 and 2, respectively.

After extracting 155 patients without social security from the main socioeconomic classes (147 from the lowest class and 8 from the middle class), a new group without health insurance coverage was formed. Comparing patients in the upper socioeconomic class, in which all individuals have a social security program, to patients without a healthcare coverage, the multivariate analysis demonstrated an improved outcome with respect to total mortality and a borderline difference for cardiac death with HRs of 0.46 (95% CI, 0.23–0.94), *P* = 0.032, and 0.37 (0.14–1.01), *P* = 0.054, respectively, whereas no difference was noted in SCD (Table 4(b)).

No gender-related differences were found between socioeconomic classes, except for a lower total mortality in females belonging to the middle and high socioeconomic groups as compared to the low socioeconomic group, with HRs of 0.10 (95% CI 0.01–0.73), *P* = 0.024, and 0.45 (95% CI 0.24–0.85), *P* = 0.014, respectively.

In the TnT positive population in the highest socioeconomic group, in which all patients were covered by a healthcare security program, total and cardiac mortality was statistically significantly lower than that in in patients without social security (HR 0.36 (95% CI, 0.15–0.83), *P* = 0.017, and 0.27 (95% CI, 0.09–0.86), *P* = 0.027, resp.), whereas SCD was not significantly affected (Table 4(b)). However, applying the same comparison to the TnT negative patients, there were no significant differences (Table 4(b)).

After extracting patients without health care coverage, total mortality and SCD still remained lower in the upper socioeconomic class, as compared to the lowest class, with HRs of 0.42 (95% CI, 0.22–0.81), *P* = 0.009, and 0.30 (95% CI, 0.10–0.93), *P* = 0.037, respectively, whereas no difference was found in cardiac death.

## 4. Discussion

A large proportion of the Argentinean population is not covered by a social security program, and this includes the city of Salta in which 60% have no coverage. In this prospective observational study, 15.8% of the admitted patients were not covered by a social security program, which is mainly due to major recruitment at the private clinics. Although the public hospital is equipped with a similar number of patient beds as the private clinics all together, this hospital only included 6.5% of the patient population, and as previously stated, the public hospital mainly attends to patients without social security.

Consequently, patients without health care insurance coverage are under-represented in our study. Therefore, we chose to divide the population into three socioeconomic classes, to assess the prognosis across a socioeconomic gradient and to compare patient groups with a social healthcare program to those without.

We found that 5-year survival from total and cardiac death increased in the upper socioeconomic group, in which all subjects had a social healthcare program, as compared to the lowest, but no difference was found in SCD. After extracting patients not covered by a social security program, there was still a significant difference in total mortality in favour of the upper socioeconomic class, and SCD (but not cardiac death) also differed significantly favoring the latter group.

Comparing the upper socioeconomic class to patients without a social security program in our multivariate analysis, in which age, gender, hypertension, DM type 2, and BNP were corrected for, a similar relationship was found for total and cardiac mortality. Thus, in our study, socioeconomic inequalities appear to be a risk factor, with or without a social security program.

In our multivariate analysis, total and cardiac mortality increased in the TnT positive patients belonging to the low socioeconomic class, whereas survival was unaffected by socioeconomic grouping in the TnT negative population. This would suggest that the low socioeconomic group with an ACS diagnosis may have received less medical attention following the index event.

We chose to use a model based on three categories instead of tertiles, due to the categorical definition of socioeconomic class, which includes both genders, whereas others have used continuous variables such as income [8] to define the socioeconomic groups in male-based studies [8, 9]. In our study 40% of the patients were females, and in the present community only a smaller part of the female population receives a regular income. It has previously been demonstrated that the incidence of sudden cardiac arrest at home or at residential institutions is higher in poorer neighbourhoods at selected sites in the USA and Canada [10, 11]. Our data support this finding. In a comparative analysis in the US and 11 western European countries, socioeconomic inequalities were found to be an essential risk factor for cardiovascular disease mortality, essentially related to cigarette smoking and excessive alcohol consumption [12]. In our study we recorded cigarette smoking but not alcohol consumption, and in the lowest socioeconomic class current smoking was less frequent as compared to the upper class, suggesting other risk factors to be of greater importance in this population.

In the Copenhagen Male Study the authors suggest that potential modifiable risk factors associated with life style and working environment are strong mediators of social inequalities in risk of ischemic heart disease [13]. In the Salta region there is a high rate of unemployment and the larger part of the workers are not protected by union rights, which may have an unfavourable effect on daily life, affecting individual health conditions.

In the Scottish Heart Health Study it was shown that prevalent [14] and incident CHD [15, 16] is related to housing tenure status (owner-occupiers or renters), regarded as a sensitive measure of social class, as house renting was predominantly a feature of the socially disadvantaged. In the lower socioeconomic class in our study, it is mainly the living conditions and not the possession of property that is influencing CHD mortality.

In the study based on socioeconomic inequalities in 22 European countries, the access to health care was found to be one of several factors influencing inequality [17]. Our findings of increased mortality in the lower socioeconomic class and among individuals without a social security program are in accordance with this statement.

An inverse relationship between education and mortality has been reported [18]. However, we did not include education in our socioeconomic model, as our study was not related to primary prevention but was based on a population with suspected coronary heart disease. Therefore, education was not regarded as an essential mediator of health in this population.

Within societies with a social democratic healthcare system in which all inhabitants receive a similar social security program, patients will receive the same medical attention irrespective of income, and in these populations health will largely depend on other factors such as education and employment. Thus, in a system with an equal social security program, primary and secondary prevention is the key to better health, whereas in a system in which 60% of inhabitants have no social security, the lack of medical attention may largely explain the increase in cardiac mortality.

Major strengths of our study is that, to our knowledge, this is the first study performed in Argentina that evaluates the socioeconomic classes and their impact on prognosis in patients admitted with chest pain and suspected ACS. Furthermore, we use strong endpoints and the baseline CHD risk factors cover a wide spectrum of variables. In addition, our socioeconomic model also includes several variables, increasing the validity and reliability of socioeconomic class definition. A primary prevention study performed in Brazil [19] concludes that there is an inverse relationship between cardiovascular mortality and income, and education and poor housing conditions. However, that is a primary prevention study with only univariate data, in contrast to our study in which data have been provided in a secondary prevention setting and correcting for potential confounders.

*Limitations*

Weaknesses of this study include the relatively small sample size (*n* = 982) compared to larger studies. A condition such as diabetes mellitus may be underestimated, as this information is mainly self-reported, and physical activity has not been accounted for. Furthermore, this study included patients from one Argentinean city, representing the demographics of the city of Salta, located in northern Argentina. Thus, our results do not necessarily represent other provinces in Argentina and should not be extrapolated to other countries. Most of our patients were included at private centers, and as only 15.8% as compared to the expected 60% had no social security coverage, this population is highly selected.

## 5. Conclusion

Although a social security program is associated with improved outcome in the high socioeconomic class as compared to patients with no social security, prognosis was mainly tied to socioeconomic inequalities in this northern Argentinean population. The effectiveness of a social security program in this community requires further attention.

## Figures and Tables

**Table 1:** General demographic indicators in Argentinean and Salta populations [3].

	Argentinean republic	Salta province/city
Population (*n*)	40,518,951	1,267,311/536,113
Men (%)	49.0	49.4/48.1
Density population (h/m^2^)	14.4	8.20/311.33
Life expectancy at birth for both sexes (years)	75.24	71.88
Life expectancy at birth for women (years)	79.10	75.24
Life expectancy at birth for men (years)	71.60	68.73
Literacy rate in population > 10 years (%)	98.1	95.6
Doctors per 10,000 inhabitants	29.8	16.8
Unemployment rate (%)	7.3	9.3
Population not covered by social work or health plan (%)	36.1	60.2
Cardiologists (*n*)	5000	98/75

**Table 2:** Baseline characteristics of the total population according to socioeconomic groups.

Characteristics, (*n*)	Low (*n* = 448)	Middle (*n* = 379)	High (*n* = 155)	*P* value
Age, years*	65.0 ± 13.6	60.6 ± 12.5	57.9 ± 13.2	<0.01
Male, *n* (%)	215 (48.0)	253 (66.8)	120 (77.4)	<0.01
Smoking status				<0.01
Current smoker, *n* (%)	79 (18.2)	99 (26.5)	60 (39.2)	
Past smoker, *n* (%)	281 (64.6)	193 (51.6)	63 (41.2)	
Never smoker, *n* (%)	75 (17.2)	82 (21.9)	30 (19.6)	
Angina pectoris, *n* (%)	116 (25.9)	75 (19.8)	32 (20.6)	0.09
CHF				
Killip class 2–4, *n* (%)	69 (15.4)	72 (19.0)	24 (15.5)	0.35
History of MI, *n* (%)	47 (10.5)	36 (9.5)	11 (7.1)	0.46
CABG, *n* (%)	25 (5.7)	15 (4.0)	7 (4.5)	0.53
PCI, *n* (%)	45 (10.0)	42 (11.1)	11 (7.1)	0.38
Hypertension, *n* (%)	303 (67.6)	243 (64.1)	88 (56.8)	0.05
History of DM 1, *n* (%)	8 (1.8)	4 (1.1)	3 (1.9)	0.62
History of DM 2, *n* (%)	83 (18.9)	84 (22.4)	20 (13.0)	0.04
STEMI, *n* (%)	52 (11.9)	66 (17.6)	26 (17.1)	0.05
TnT release, *n* (%)	168 (37.5)	157 (41.5)	63 (40.6)	0.48
eGFR (*μ*mol L^−1^)*	80.3 ± 29.2	81.7 ± 28.2	83.0 ± 26.6	0.55
Cholesterol/use of statin^†^, *n* (%)	81 (18.1)	52 (13.7)	27 (17.4)	0.22
Use of beta blockers, *n* (%)	121 (27.6)	96 (25.6)	36 (23.4)	0.57
CHD, *n* (%)	163 (36.9)	120 (31.7)	41 (26.5)	0.04
BMI (kg/m^2^)*	28.2 ± 4.8	27.9 ± 4.2	28.2 ± 3.9	0.60
25(OH)D (nM)*	50.2 ± 18.2	51.5 ± 15.8	53.1 ± 15.3	0.17
BNP Quartiles				<0.01
Q1	94 (21.0)	93 (24.5)	58 (37.4)	
Q2	92 (20.5)	106 (28.0)	48 (31.0)	
Q3	121 (27.0)	94 (24.8)	31 (20.0)	
Q4	141 (31.5)	86 (22.7)	18 (11.6)	
hsCRP Quartiles				0.12
Q1	111 (24.8)	88 (23.4)	46 (29.7)	
Q2	99 (22.1)	100 (26.6)	48 (31.0)	
Q3	119 (26.6)	95 (25.3)	30 (19.4)	
Q4	118 (26.4)	93 (24.7)	31 (20.0)	

^†^Concentration > 250 mg/dL.

*Mean ± SD.

SD: standard deviation; 25(OH)D: 25-hydroxyvitamin D; CHF: congestive heart failure; MI: myocardial infarction; CABG: coronary artery bypass grafting; PCI: percutaneous coronary intervention; DM: diabetes mellitus; STEMI: ST-elevation myocardial infarction; TnT: troponin T; eGFR: estimated glomerular filtration rate; CHD: coronary heart disease; BMI: body mass index; BNP: B-type natriuretic peptide; hsCRP: high sensitivity C-reactive protein.

**Table 3:** Baseline characteristics for patients with and without social security.

Characteristics	Without social security (*n* = 155)	With social security (*n* = 827)	*P* value
Age, years*	66.3 ± 13.3	61.4 ± 13.3	<0.01
Male, *n* (%)	98 (63.2)	490 (59.3)	0.35
Smoking status			0.00
Current smoker, *n* (%)	23 (15.2)	215 (26.5)	
Past smoker, *n* (%)	104 (68.9)	433 (53.4)	
Never smoker, *n* (%)	24 (15.9)	163 (20.1)	
Angina pectoris, *n* (%)	41 (26.5)	182 (22.0)	0.23
CHF			
Killip class 2–4, *n* (%)	26 (16.8)	139 (16.8)	0.99
History of MI, *n* (%)	21 (13.5)	73 (8.8)	0.07
CABG, *n* (%)	8 (5.2)	39 (4.8)	0.81
PCI, *n* (%)	16 (10.3)	82 (9.9)	0.88
Hypertension, *n* (%)	92 (59.4)	542 (65.5)	0.14
History of DM 1, *n* (%)	2 (1.3)	13 (1.6)	0.79
History of DM 2, *n* (%)	27 (17.6)	160 (19.6)	0.57
STEMI, *n* (%)	20 (13.2)	124 (15.3)	0.50
TnT release, *n* (%)	67 (43.2)	321 (38.9)	0.31
eGFR (*μ*mol L^−1^)*	81.5 ± 30.0	81.2 ± 28.1	0.91
Cholesterol/use of statin^†^, *n* (%)	29 (18.7)	131 (15.8)	0.38
Use of beta blockers, *n* (%)	36 (23.8)	217 (26.6)	0.49
CHD, *n* (%)	58 (37.9)	266 (32.4)	0.18
BMI (kg/m^2^)*	28.1 ± 3.9	28.1 ± 4.5	0.93
25(OH)D (nM)*	49.8 ± 19.4	51.4 ± 16.4	0.27
BNP Quartiles			<0.01
Q1	20 (12.9)	225 (27.2)	
Q2	26 (16.8)	220 (26.6)	
Q3	48 (31.0)	198 (23.9)	
Q4	61 (39.4)	184 (22.2)	
hsCRP Quartiles			0.00
Q1	35 (22.6)	210 (25.5)	
Q2	24 (15.5)	223 (27.1)	
Q3	45 (29.0)	199 (24.2)	
Q4	51 (32.9)	191 (23.2)	

^†^Concentration > 250 mg/dL.

*Mean ± SD.

SD: standard deviation; 25(OH)D: 25-hydroxyvitamin D; CHF: congestive heart failure; MI: myocardial infarction; CABG: coronary artery bypass grafting; PCI: percutaneous coronary intervention; DM: diabetes mellitus; STEMI: ST-elevation myocardial infarction; TnT: troponin T; eGFR: estimated glomerular filtration rate; CHD: coronary heart disease; BMI: body mass index; BNP: B-type natriuretic peptide; hsCRP: high sensitivity C-reactive protein.

**Table:** (a)

Mode of death at 5 y FU		Low	Middle	High	HR (95% CI)	*P* value
All patients						
AC mortality, *n* (%)		102 (22.8)	57 (15.0)	14 (9.0)	0.46 (0.26–0.83)	0.009
C mortality, *n* (%)		55 (12.3)	29 (7.7)	8 (5.2)	0.42 (0.19–0.94)	0.035
SCD, *n* (%)		36 (8.0)	18 (4.7)	5 (3.2)	0.47 (0.18–1.23)	0.123

	Without social security				*HR (95% CI)	

All patients						
AC. mortality, *n* (%)	38 (24.5)	64 (21.3)	57 (15.4)	14 (9.0)	0.46 (0.25–0.88)	0.018
C. mortality, *n* (%)	24 (15.5)	31 (10.3)	29 (7.8)	8 (5.2)	0.38 (0.16–0.89)	0.027
SCD, *n* (%)	15 (9.7)	21 (7.0)	18 (4.9)	5 (3.2)	0.46 (0.16–1.26)	0.132
TnT positive patients						
AC. mortality, *n* (%)	24 (35.8)	36 (35.0)	42 (27.1)	10 (15.9)	0.49 (0.23–1.06)	0.069
C. mortality, *n* (%)	15 (22.4)	16 (15.5)	22 (14.2)	6 (9.5)	0.45 (0.16–1.25)	0.126
SCD, *n* (%)	8 (11.9)	13 (12.6)	13 (8.4)	4 (6.3)	0.73 (0.22–2.47)	0.618
TnT negative patients						
AC. mortality, *n* (%)	14 (15.9)	28 (14.1)	15 (7.0)	4 (4.3)	0.38 (0.12–1.17)	0.092
C. mortality, *n* (%)	9 (10.2)	15 (7.6)	7 (3.3)	2 (2.2)	0.28 (0.06–1.28)	0.100
SCD, *n* (%)	7 (8.0)	8 (4.0)	5 (2.3)	1 (1.1)	0.18 (0.02–1.45)	0.106

HR (95% CI): hazard ratio (95% confidence interval).

HRs for low as compared to high socioeconomic group are depicted in the first section of the table. The lower section of the table shows the *HRs for patients without social security as compared to the high socioeconomic group, in which all patients had a social security program.

Variables included in the multivariate model are gender and age.

AC: all cause, C: cardiac, SCD: sudden cardiac death, 5 y: 5 years, FU: follow-up.

**Table:** (b)

Mode of death at 5 y FU		Low	Middle	High	HR (95% CI)	*P* value
All patients						
AC. mortality, *n* (%)		102 (22.8)	57 (15.0)	14 (9.0)	0.42 (0.22–0.80)	0.008
C. mortality, *n* (%)		55 (12.3)	29 (7.7)	8 (5.2)	0.39 (0.15–0.99)	0.047
SCD, *n* (%)		36 (8.0)	18 (4.7)	5 (3.2)	0.37 (0.13–1.06)	0.065

	Without social security				*HR (95% CI)	

All patients						
AC. mortality, *n* (%)	38 (24.5)	64 (21.3)	57 (15.4)	14 (9.0)	0.46 (0.23–0.94)	0.032
C. mortality, *n* (%)	24 (15.5)	31 (10.3)	29 (7.8)	8 (5.2)	0.37 (0.14–1.01)	0.054
SCD, *n* (%)	15 (9.7)	21 (7.0)	18 (4.9)	5 (3.2)	0.47 (0.15–1.53)	0.211
TnT positive patients						
AC. mortality, *n* (%)	24 (35.8)	36 (35.0)	42 (27.1)	10 (15.9)	0.36 (0.15–0.83)	0.017
C. mortality, *n* (%)	15 (22.4)	16 (15.5)	22 (14.2)	6 (9.5)	0.31 (0.10–0.98)	0.046
SCD, *n* (%)	8 (11.9)	13 (12.6)	13 (8.4)	4 (6.3)	0.56 (0.13–2.36)	0.430
TnT negative patients						
AC. mortality, *n* (%)	14 (15.9)	28 (14.1)	15 (7.0)	4 (4.3)	0.45 (0.13–1.63)	0.224
C. mortality, *n* (%)	9 (10.2)	15 (7.6)	7 (3.3)	2 (2.2)	0.28 (0.03–2.30)	0.233
SCD, *n* (%)	7 (8.0)	8 (4.0)	5 (2.3)	1 (1.1)	0.27 (0.03–2.38)	0.240

HR (95% CI): hazard ratio (95% confidence interval).

HRs for low as compared to high socioeconomic group are depicted in the first section of the table. The lower section of the table shows the *HRs for patients without social security as compared to the high socioeconomic group, in which all patients had a social security program.

Variables potentially included in the stepwise multivariate model: gender, age, smoking, hypertension, index diagnosis, DM, CHF, history of previous CHD, hypercholesterolemia/use of statins, TnT > 0.01 ng/mL, eGFR, hsCRP, BNP, 25(OH)D, body mass index (kg/m^2^), months of sampling, and beta blockers prior to enrolment.

AC: all cause, C: cardiac, SCD: sudden cardiac death, 5 y: 5 years, FU: follow-up. DM: diabetes mellitus, CHF: congestive heart failure, CHD: coronary heart disease, eGFR: estimated glomerular filtration rate, hsCRP: high sensitivity C reactive protein, BNP: brain natriuretic peptide, 25(OH)D: vitamin D.
